# Effects of high temperature on different restorations in forensic identification: Dental samples and mandible

**DOI:** 10.4103/0974-2948.71056

**Published:** 2010

**Authors:** Kalpana A Patidar, Rajkumar Parwani, Sangeeta Wanjari

**Affiliations:** *Department of Oral and Maxillofacial Pathology, Modern Dental College and Research Center, Airport Road, Gandhi Nagar, Indore, Madhya Pradesh, India*

**Keywords:** Dental restoration, forensic identification, glass ionomer cement (GIC), metal ceramic crown, Ni-Cr metal crown, temperature, zinc phosphate cement [Zn3(PO4)2]

## Abstract

**Introduction:**

The forensic odontologist strives to utilize the charred human dentition throughout each stage of dental evaluation, and restorations are as unique as fingerprints and their radiographic morphology as well as the types of filling materials are often the main feature for identification. The knowledge of detecting residual restorative material and composition of unrecovered adjacent restoration is a valuable tool-mark in the presumptive identification of the dentition of a burned victim. Gold, silver amalgam, silicate restoration, and so on, have a different resistance to prolonged high temperature, therefore, the identification of burned bodies can be correlated with adequate qualities and quantities of the traces. Most of the dental examination relies heavily on the presence of the restoration as well as the relationship of one dental structure to another. This greatly narrows the research for the final identification that is based on postmortem data.

**Aim:**

The purpose of this study is to examine the resistance of teeth and different restorative materials, and the mandible, to variable temperature and duration, for the purpose of identification.

**Materials and Methods:**

The study was conducted on 72 extracted teeth which were divided into six goups of 12 teeth each based on the type of restorative material. (Group 1 - unrestored teeth, group 2 - teeth restored with Zn_3_(PO_4_)_2_, group 3 - with silver amalgam, group 4 with glass ionomer cement, group 5 - Ni-Cr-metal crown, group 6 - metal ceramic crown) and two specimens of the mandible. The effect of incineration at 400°C (5 mins, 15 mins, 30 mins) and 1100°C (15 mins) was studied.

**Results:**

Damage to the teeth subjected to variable temperatures and time can be categorized as intact (no damage), scorched (superficially parched and discolored), charred (reduced to carbon by incomplete combustion) and incinerated (burned to ashes).

## Introduction

Historically teeth and dental materials have been studied to aid the identification process of human remains. Forensic odontology in particular has been seen to be useful when the damage has been caused by heat.[1–3]

Human identification is one of the major fields of study in forensic science because it deals with the human body and aims at establishing human identity. Dental identification is one of the most reliable and frequently applied methods of identification, and forensic odontology is a speciality in itself. The establishment of forensic odontology is a unique discipline that has been attributed to Dr. Oscar Amoedo (Father of Forensic Odontology) who identified the victims of fire accident in Paris, France in 1897.[4]

At times, fire in a building may prove to be a wild fire and can easily go out of control, with devastating, fatal effects. Fire remains one of the major causes of morbidity and mortality throughout the world and identification of a body from the fatal fire remains a daunting task.[5] Norrlander[6] classified body burns into five categories: (1) superficial burns, (2) destroyed epidermis areas, (3) destruction of the epidermis, dermis, and necrotic areas in the underlying tissues, (4) total destruction of the skin and deep tissue, and (5) burned remains.

Identification of human remains in mass disasters is a difficult task. Identification of burned bodies starts with the objects that have remained with the body. Teeth are considered to be the most indestructible components of the human body. Teeth have the highest resistance to most environmental effects like fire, desiccation, and decomposition. Teeth survive most natural disasters and provide a positive, personal identification of an otherwise unrecognizable body. It begins with the correlation of dental records to observed restorations.[7] As the destruction of the burned victims of the third, fourth, and fifth categories is extensive, such remains cannot be identified and odontologists are called to assist in the identification.[4,8]

In recent years, dentistry has been benefited from a marked increase in the development of esthetic materials. However, the usefulness of traditional materials has not been eliminated.[9] Forensic odontology is concerned with the identification of different restorative materials subjected to variable temperatures, which provide important post-mortem clues with the availability of premortem records.[10] In cases of mass disasters associated with fire, identification of the burned victims can be a real challenge to the forensic team.[11] It is hypothesized that a systematic approach toward the inspection of the restoration of teeth and fractured bone after the burn can ensure maximum data and help in identification of the burned body.

## Aims and Objectives

1. To subject the teeth and different restorative materials, and the mandible to different temperatures and time of exposure
2. To assess the degree of destruction in color, shape, and structure of the teeth, restoration, and mandible after incineration
3. To broaden the knowledge of resistance of the teeth, restorative material, and mandible to different temperatures and durations
4. To evaluate the findings of the study with regard to its application to forensic identification

## Materials and Methods

In the present study, 72 extracted teeth were collected from the Department of Oral and Maxillofacial Surgery, in the Modern Dental College and Research Center, Indore. Teeth with decay, abrasion, erosion, hypoplasia, Fracture and / or restorations were excluded from the study. Two specimens of the mandible — as remains of unknown adult bodies — were also included in the study.

The 72 teeth were divided into six groups of 12 teeth each — Group 1 Unrestored teeth, Group 2 — Teeth restored with Zn_3_(PO_4_)_2_ (DeTrey® Zinc Dentsply, DeTrey GmbH, Konstanz, Germany), Group 3 — Teeth restored with silver amalgam (DPI Alloy fine grain, Silver Tin Dental Amalgam Alloy, Bombay Burmah Trading Corporation Ltd., India), Group 4 — Teeth restored with Glass Ionomer Cement (GIC) (ChemFlex^™^, Dentsply DeTrey GmbH, Konstanz, Germany), Group 5 — Teeth with Ni–Cr metal crown (Ruby max white metal soft type, Ruby Dental Products Osaka Japan), and Group 6 — Teeth with a metal ceramic crown (Ceramic powder-Cermico 3 Dentsply USA, Metal-Max bond Ruby Dental Products, Osaka, Japan). Two specimens of the mandible were labeled and both restored by the same operator: Specimen 1 — Containing six teeth restored with silver amalgam, Zn_3_(PO_4_)_2_, GIC, Ni–Cr metal crown, metal-ceramic crown, and Specimen 2 — containing four teeth restored with silver amalgam, GIC, Zn_3_(PO_4_)_2_, and Ni–Cr metal crown. All the restored cavities were class I.

Four plates containing teeth of different groups were prepared by an investment material of approximately equal dimension and numbered as — Plate I, Plate 2, Plate 3, and Plate 4: the roots of the unrestored and restored teeth were kept totally immersed in the investment material, with the aim of simulating the real oral cavity. Each plate contained teeth arranged in six columns, with each column made up of three teeth so that each plate contained eighteen teeth. All plates contained a first column of unrestored teeth, a second column of teeth restored with Zn_3_(PO_4_)_2_, a third column of teeth restored with silver amalgam, a fourth column of teeth restored with GIC, a fifth column of Ni–Cr metal crown, and a sixth column of metal ceramic crown.

Plate I was incinerated at 400°C for five minutes, Plate II was incinerated at 400°C for 15 minutes, Plate III was incinerated at 400°C for 30 minutes, and Plate IV at 1100°C for 15 minutes. Specimen 1 of the mandible was incinerated at 400°C for 15 minutes and Specimen 2 was incinerated at 1100°C for 15 minutes.

## Results

The effect of varying temperatures on the unrestored teeth was observed mainly in the form of color change, ranging from brown and black to gray. At the highest temperature they showed an ashy appearance and fragmentation.

Teeth restored with Zinc phosphate cement mainly showed discoloration, cracks, fissures, and finally disintegration at the highest temperature.

Sliver amalgam initially showed loss of marginal seal and discoloration, followed by globules of restorations at the highest temperature.

GIC mainly showed discoloration, cracks and fractures.

Ni–Cr metal crowns initially showed loss of glaze with the crown in place, but at the highest temperature the crowns were dislodged.

Ceramic crowns did not show any changes initially, except loosening, but at 1100°C they showed a loss of morphology.

The mandible, at 400°C, showed blackening and fracture, but at 1100°C it was totally ashen, gray, and shrunken.

The results of the study have been computed in the tables [Tables 1–7]

**Table 1:** Effect of different temperatures and time on unrestored teeth [Figure 1]

Temperature	Time	Effect
400°C	5 minutes	Little brownish discoloration of teeth
400°C	15 minutes	Blackish-brown teeth
400°C	30 minutes	Black charcoal gray teeth with slacky appearance
1100°C	15 minutes	Grayish-white ashy appearance, with multiple cracks resulting in fragments of teeth mixed indiscriminately

**Figure 1:**
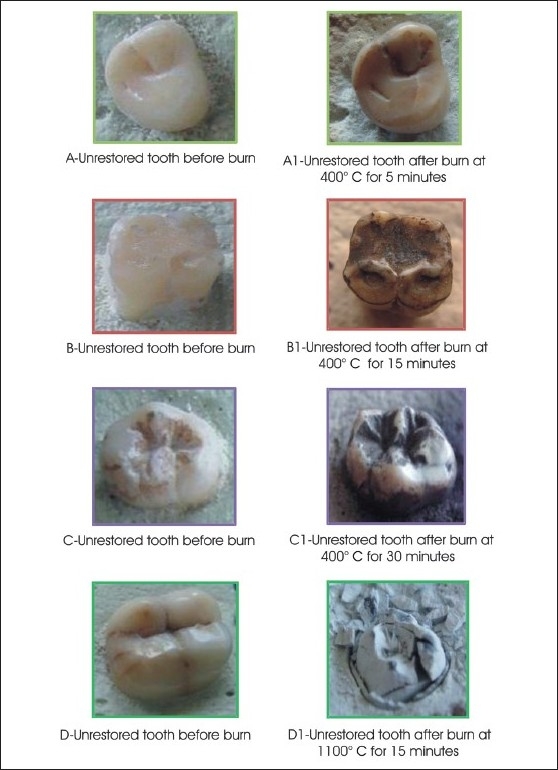
Effects of different temperature and time on unrestored teeth

**Table 2:** Effect of different temperatures and time on teeth restored with Zn3(PO4)2 [Figure 2]

Temperature	Time	Effect
400°C	5 minutes	Shrinkage and gap between margins to a small extent without any discoloration
400°C	15 minutes	Blackish discoloration easily visible marginal shrinkage. Surface appearing dry and little rough
400°C	30 minutes	Charring of restoration, remarkable unconfined margins and shaggy surface
1100°C	15 minutes	Fragile teeth shredded down with disordered restoration, ashen grayish appearance, with remarkable discontinuation of margins

**Figure 2:**
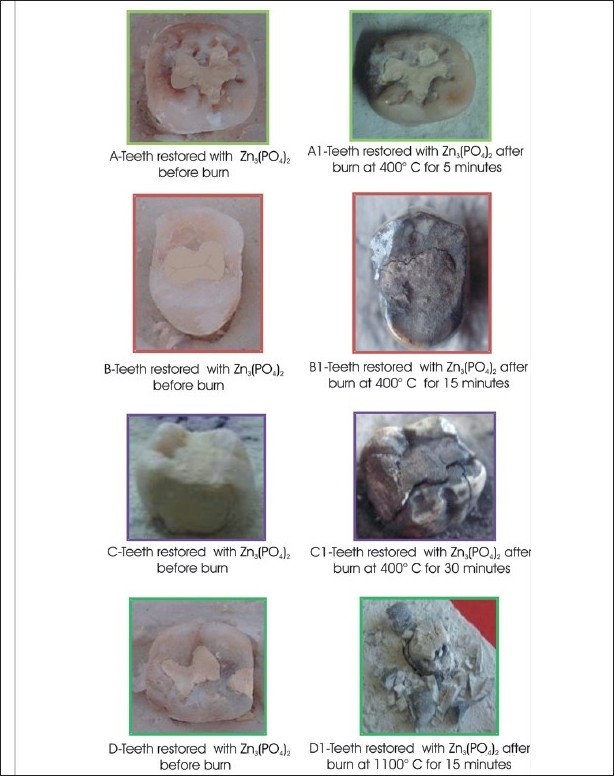
Effects of different temperature and time on teeth restored with Zn3(PO4)2

**Table 3:** Effect of different temperatures and time on teeth restored with silver amalgam [Figure 3]

Temperature	Time	Effect
400°C	5 minutes	Striking expansion resulting in bulging out of the free surface that is with a rough surface and loss of cohesion
400°C	15 minutes	Noticeable expansion, plumped-out appearance, loss of marginal contour and contact
400°C	30 minutes	Rough coarse uneven surface. Multiple cracks, overflow and expansion leading to unconfined and discontinuous margins
1100°C	15 minutes	Identifiable spherical globules of restoration, showing grayish to whitish discoloration and splintered teeth

**Figure 3:**
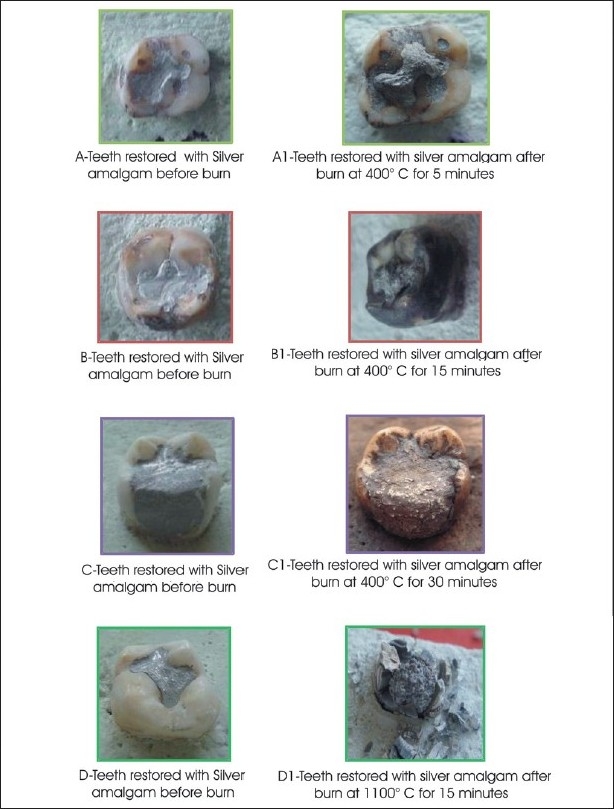
Effects of different temperature and time on teeth restored with silver amalgam

**Table 4:** Effect of different temperatures and time on teeth restored with GIC [Figure 4]

Temperature	Time	Effect
400°C	5 minutes	Loss of shiny surface and blackish gray discoloration of restoration and marginal shrinkage
400°C	15 minutes	Charcoal gray discoloration with roughened surface
400°C	30 minutes	Shrinkage and crack in restoration resulting in uncontended margins
1100°C	15 minutes	Restorations unsupported by teeth, resulting in discretion (separate) of restoration. Fractured restoration

**Figure 4:**
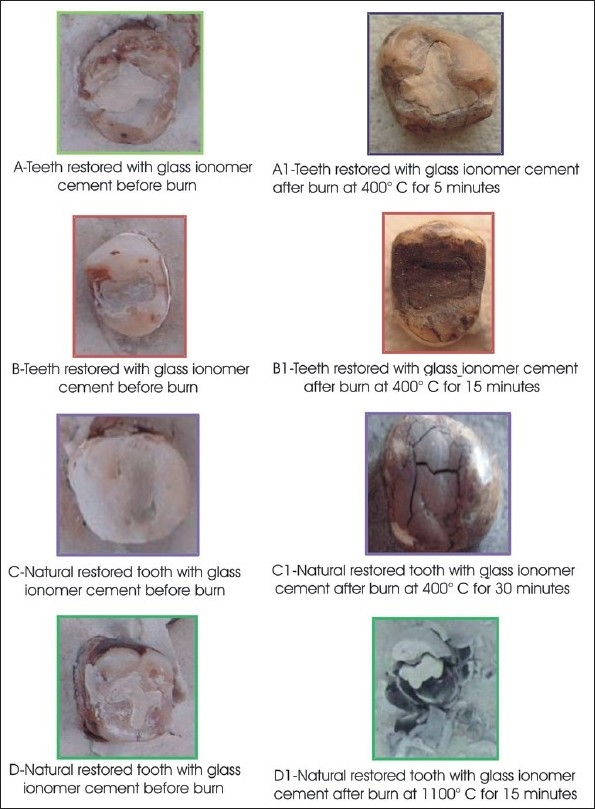
Effects of different temperature and time on teeth restored with glass ionomer cement

**Table 5:** Effect of different temperature and time on teeth restored with NI–Cr metal crown [Figure 5]

Temperature	Time	Effect
400°C	5 minutes	Little loss of glaze
400°C	15 minutes	Loss of glaze and clearly perceptible disintegration of luting cement, resulting in loss of marginal seal at the cervical area
400°C	30 minutes	Blackened, glossy, and shiny surface. Crown could be displaced
1100°C	15 minutes	Rough, crumpled surface and decomposed core leading to a dislodged crown

**Figure 5:**
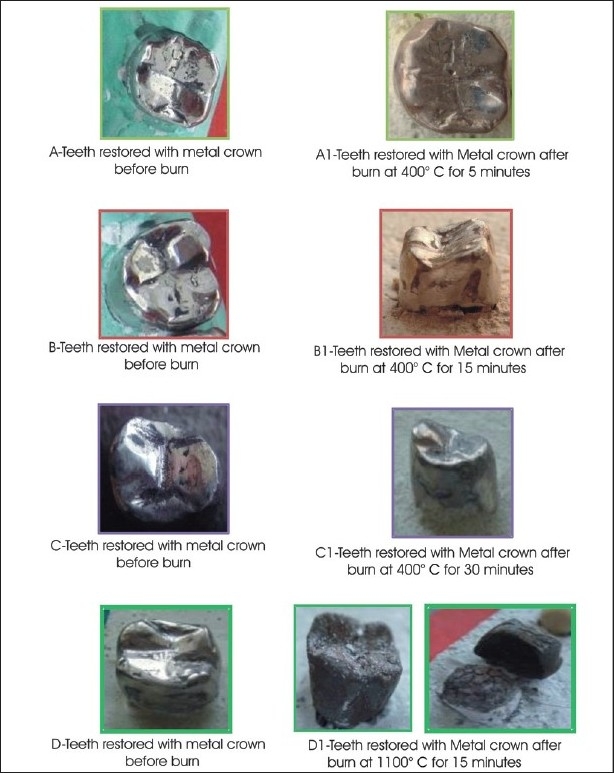
Effects of different temperature and time on teeth restored with Ni-Cr metal crown

**Table 6:** Effect of different temperatures and time on teeth restored with ceramic crown [Figure 6]

Temperature	Time	Effect
400°C	5 minutes	Loss of continuum depicted by unrestrained margins of the crown with teeth. Loosening of crown without much change in color or texture
400°C	15 minutes	Not many changes in proper ceramic. Indistinct margins resulting in shifting of crown
400°C	30 minutes	Pitted surface with slight discoloration. Easily observable creased core resulting in displaced crown
1100°C	15 minutes	Overflowingd ceramic with loss of morphology, changes of glaze texture to uneven patchy pattern. Sometimes exhausted core resulting in displaced / exfoliated crown

**Figure 6:**
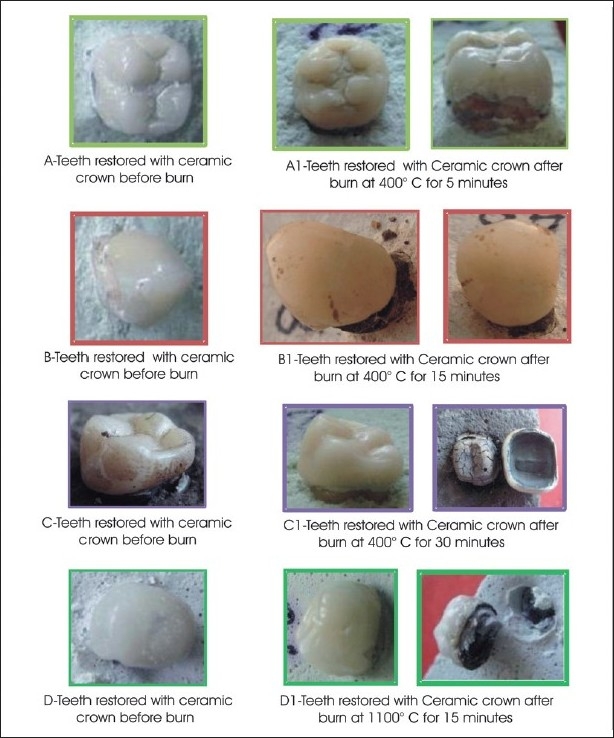
Effects of different temperature and time on teeth restored with metal-ceramic crow

**Table 7:** Effect of different temperatures and time on the mandible specimen [Figure 7]

Specimen	Temperature	Time	Effect
1	400°C	15 minutes	Carbonization of mandible resulting in granular black surface. Vertical curved transverse fracture, typical of heat exposure and thinning out of cortical plates.
2	1100°C	15 minutes	Gross shrinkage, deformation of mandible, ashen gray appearance, and multiple fractures.

**Figure 7:**
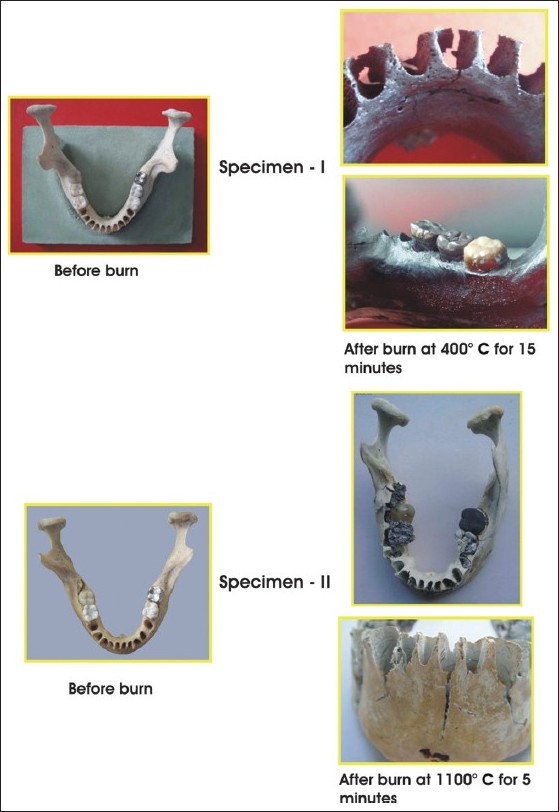
Effects of different temperature and time on the specimens of mandibles

## Discussion

Forensic medicine works for forensic identification. By its nature it is a multidisciplinary team effort relying on positive identification methodologies. In forensic odontology a great deal of effort goes into identifying the victim. One method of identification in forensic odontology is to examine the burned bodies and their fine traces, as well as to examine the resistance of teeth and restorative material to high temperature.[5]

In 1897, an article entitled, ‘The role of a dentist in the identification of the victim of the catastrophe of Bazar de la Charité, Paris,’ 4 May, 1897, was presented by Dr. Oscar Amoedo (Professor at the dental school in Paris) at the international medical congress of Moscow. The Bazar at which the wealthy women of Paris annually raised money for projects for the poor was destroyed within 10 minutes and 126 people lost their lives. The bodies of those killed by the fire were brought to the Industrial Palace for identification. Visual identification was difficult because many were mutilated and extensively burned. Recognition was made by body remains. When 30 remaining corpses could not be identified, the Paraguayan consul called a dentist to identify the burned bodies and dental identification was carried out through the fire remains.[12]

In our study we have observed the visual damage to the unrestored and restored teeth as well as mandible due to fire.

In our research, the unrestored teeth mainly showed a color change from brown to black to gray, which turned completely ashy-white at 1100°C. This is directly related to the level of carbonization and incineration of teeth. All of these changes were also described by Merlati *et al*,[13,14] by Gunther and Schdmidt-quoted by Rotzscher[5] Horsanyi L[10] 1975, Muller M[15] *et al*, 1998, and Merlati G, Danesino P[14] *et al*, 2002. Thus, small fragments of teeth can be identified from the burn remains and a reliable estimation of the temperature of exposure can be made.

Silver amalgam in our study initially (at 400°C for five minutes) showed loss of glaze, expansion, and finally at 1100°C, globule formation and splintering. Similar changes were observed by Merlati[13,14] and Gunther and Schdmidt. These globules in the restorations could be due to alloy dissociation, where the mercury evaporates through the gaseous bubbles, which form blisters or nodules.[13] Gunther and Schmidt[5] called these silver globules ‘Silver bullets’. Merlati G and Savio C,[13] 2004, studied the effect of predetermined temperature on amalgam restorations and found that restorations at different temperature levels remained in place and maintained their shape, despite disintegration of the crowns.

Our research indicated that teeth restored with GIC showed discoloration, cracks, and fractures as shown by Rossouw RS[16] *et al*, 1999. We would like to emphasize that these remains of the restorations are important for the purpose of identification, as they are fire-resistant and radio opaque.

Zn_3_(PO_4_)_2_ mainly showed shrinkage and discoloration at 400°C, to an ashen-gray appearance at 1100°C. The pattern of the crack on the surface of restorations can assist in the type of heat exposure and help to trace the origin of the fire.

Ni—Cr, and metal ceramic crowns initially showed loss of glaze and finally a slight loss of morphology, with loosening of the crowns at 1100°C. We would like to emphasize that some type of porcelain alloys have a temperature of melting from 1,288°C to 1,371°C. This is the main advantage of porcelain, which is responsible for its wide acceptance as a restorative material, as it has high strength and high resistance to wear and tear. In fact it provides such hardness that at times it complicates the occlusal adjustment, and laboratory labor is more expensive than its clinical manipulation. Such restorations are a boon not only to restorative dentistry, but also to forensic dentistry. Therefore, the premortem data and fire remains of these restorations can be of great help when resolving the daunting task of identifying a body from a fatal fire.[17]

We had incinerated a specimen mandible at 400°C for 15 minutes. The mandible was totally carbonized and typical transverse fractures were observed, whereas, another specimen was incinerated at 1100°C for 15 minutes and gross shrinkage and ashen gray discoloration, with multiple fractures occurred.

The results of the mandible specimens versus the unrestored and restored teeth were compared and similar observations were reported. We thought it could be due to the new methods used, with the roots of the teeth kept totally immersed in the investment material before the burning test. Thus, it seems possible to consider this new method as reliable and a good experimental simulation of the real oral cavity.

However, it has to be pointed out that height and weight muscles may prove unreliable in human identification because of the drying of tissues. A skeleton may be a great asset, but as bones are subjected to heat, fractures occur, due to the action of dehydration on the bony collagen. When the elasticity of the bone is reduced it undergoes shrinkage deformation and the distortion results in a fracture. Similar results were observed in our study and some patterns of the fractures were typical to heat, and assisted in tracing the origin of the fire.

From these, observable damages of the teeth subjected to variable temperatures and time can be categorized as Intact (no damage), Scorched (Superficially parched and discolored), Charred (Reduced to carbon by incomplete combustion), and Incinerated (Burned to ashes).

The results of our research provide valuable information about the difference in thermal stability of various restorative materials and the mandible. The results clearly indicate that as the temperature increases the rate of decomposition of the restorative material also increases. The resistance of restoration to variable temperature is unique in itself. There is deformation, loss of elasticity, carbonization, and fracture in the bones.

It can be stated that along with the fire remains, effects on restorative materials and bone should arm the clinician with additional means of narrowing the possibilities of positive determination. Utilizing methods to access the fire remains will prevent loss of potential dental records, on condition that dental records of all the restorations are maintained accurately.

## Conclusion

Forensic dental identification of the victims of fires is often a complex and challenging endeavor. Utilizing knowledge of charred human dentition and residues of restorative material can help in the recognition of bodies burned beyond recognition. It is hoped that this study can imprint the importance of the preplanned and systematic approach toward the preservation of charred dentition, as at times it could prove to be the best evidence for identification of those who are extensively burned.
